# Acute Cerebellar Inflammation and Related Ataxia: Mechanisms and Pathophysiology

**DOI:** 10.3390/brainsci12030367

**Published:** 2022-03-10

**Authors:** Md. Sorwer Alam Parvez, Gen Ohtsuki

**Affiliations:** 1 Department of Drug Discovery Medicine, Kyoto University Graduate School of Medicine, Kyoto 606-8397, Japan; sorwersust@yahoo.com; 2 Department of Genetic Engineering & Biotechnology, Shahjalal University of Science & Technology, Sylhet 3114, Bangladesh

**Keywords:** cerebellum, acute inflammation, ataxia, infection, microglia, immune-triggered plasticity, acute cerebellitis, inflammatory mediators, Ca^2+^-activated K^+^ channels

## Abstract

The cerebellum governs motor coordination and motor learning. Infection with external microorganisms, such as viruses, bacteria, and fungi, induces the release and production of inflammatory mediators, which drive acute cerebellar inflammation. The clinical observation of acute cerebellitis is associated with the emergence of cerebellar ataxia. In our animal model of the acute inflammation of the cerebellar cortex, animals did not show any ataxia but hyperexcitability in the cerebellar cortex and depression-like behaviors. In contrast, animal models with neurodegeneration of the cerebellar Purkinje cells and hypoexcitability of the neurons show cerebellar ataxia. The suppression of the Ca^2+^-activated K^+^ channels in vivo is associated with a type of ataxia. Therefore, there is a gap in our interpretation between the very early phase of cerebellar inflammation and the emergence of cerebellar ataxia. In this review, we discuss the hypothesized scenario concerning the emergence of cerebellar ataxia. First, compared with genetically induced cerebellar ataxias, we introduce infection and inflammation in the cerebellum via aberrant immunity and glial responses. Especially, we focus on infections with cytomegalovirus, influenza virus, dengue virus, and SARS-CoV-2, potential relevance to mitochondrial DNA, and autoimmunity in infection. Second, we review neurophysiological modulation (intrinsic excitability, excitatory, and inhibitory synaptic transmission) by inflammatory mediators and aberrant immunity. Next, we discuss the cerebellar circuit dysfunction (presumably, via maintaining the homeostatic property). Lastly, we propose the mechanism of the cerebellar ataxia and possible treatments for the ataxia in the cerebellar inflammation.

## 1. Introduction

The cerebellum governs motor coordination and motor learning [1,2,3], while recent views indicated the cerebellum is involved in not only involuntary movement but also various cognitive activities [4,5,6,7,8], interacting with multiple brain regions via projection and innervation of the compartments of the cerebellum [9,10,11,12,13,14,15,16,17,18,19,20]. One of the essences of the circuit function is hypothesized to be the compensation of error signals of the external sensory input and the internal model [21,22]. Thus, the cerebellum could be regarded as a universal processor for sensory acquisition and internal models, and its association with synaptic and non-synaptic plasticity could be the biological correlates of higher-order cognition [3,8,23,24,25,26,27,28].

Infection with external microorganisms, such as viruses, bacteria, and fungi, induces acute cerebellar inflammation. Those microorganisms cause severe inflammation when entering the brain via the innate immune system initially and its receptors, pattern recognition receptors, which specifically recognize molecules of pathogens as PAMPs (Pathogen-Associated Molecular Patterns), and molecules released by damaged cells as DAMPs (Damage-Associated Molecular Patterns) [29]. Patients with inflammation in the cerebellum show the symptom of acute cerebellar inflammation, including ataxia.

**Figure 1:**
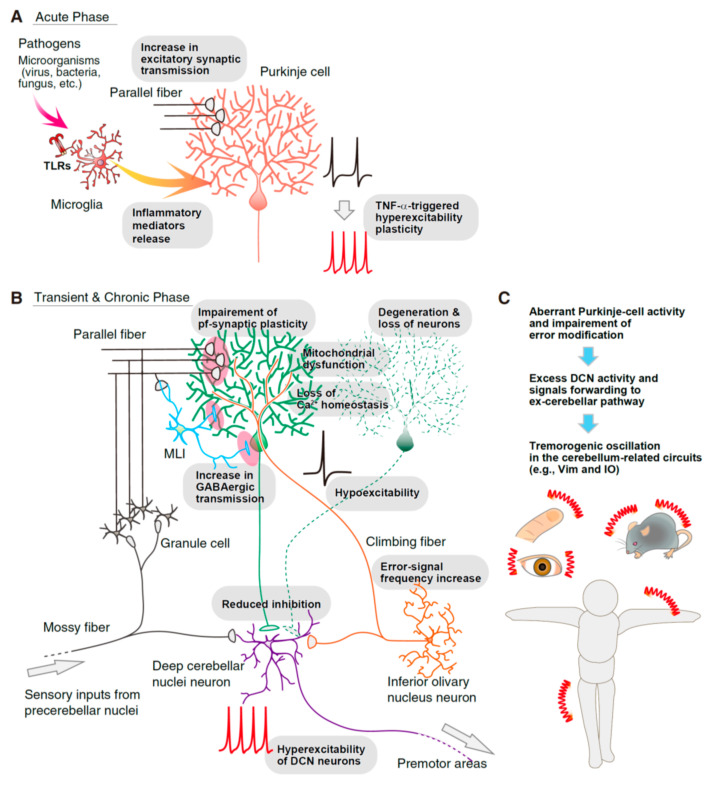
Transition of the cerebellar dysfunction in the infection model. (**A**) In response to invaded pathogens (e.g., virus, bacteria, fungus, etc.), as the acute phase, microglia present innate immune responses and release inflammatory cytokines (e.g., TNF-α, IL-1β, IL-6, etc.), which increase the intrinsic excitability of Purkinje cells in the cerebellar cortex and modulate the presynaptic release and postsynaptic responsiveness of the excitatory synapses [30]. (**B**) In the transient phase or chronic phase of the infection, Purkinje cells show various disruptions of the physiological properties. In the drawing, we summarized possible dysfunctions: an impairment of the parallel-fiber’s long-term synaptic plasticity and reduced parallel-fiber innervation, excess climbing-fiber innervations, an increase in GABAergic synaptic transmission, loss of Ca^2+^ homeostasis of Purkinje cells, hypoexcitability and aberrant oscillation, mitochondrial dysfunction, degeneration and loss of Purkinje cells, resultant reduction in the inhibitory input to deep cerebellar nuclei (DCN) neurons, and hyperexcitability of DCN neurons. (**C**) Aberrant Purkinje-cell activity and impairment of error modification cause the excess DCN activity, forwarding it to the efferent cerebellar pathways that contribute to the generation of tremor/ataxia.

In this review, we would like to discuss the acute cerebellar inflammation caused by infection and aberrant immunity (Figure 1). In a recent model of acute inflammation in the cerebellar cortex, rodents showed multiple depressive-like behaviors associated with hyperexcitability of Purkinje cells [30]. Transient exposure to lipopolysaccharide (LPS: an outer component of Gram-negative bacteria) of the brain regions and activated microglia induced prominent modulation of synaptic and non-synaptic activity across various types of neurons in the cerebellum, neocortex, hippocampus, striatum, and spinal cord [30,31,32,33,34,35,36,37] (Figure 1A), which may disrupt behaviors and cause disease-like symptoms. In the cerebellar Purkinje cells, acute inflammation by LPS or heat-killed Gram-negative bacteria induces hyperexcitability through akin signaling of the intrinsic plasticity. There, small conductance Ca^2+^-activated K^+^-channel (SK channel) function is downregulated [38,39,40,41,42,43], although the animals do not show any ataxia/tremor [30]. In the cerebellum with exposure to LPS, both presynaptic release and postsynaptic responsiveness of the excitatory synaptic transmission of Purkinje cells induces the increase in both amplitude and frequency from the recording of spontaneous and miniature excitatory postsynaptic currents (EPSCs) [30]. In contrast, the injection of the SK channel blocker, apamin, into the cerebellum induced ataxic behavior. These results suggest that microglia-triggered hyperexcitability of Purkinje cells is not sufficient for eliciting cerebellar ataxia, while chronic and neurodegenerative processes would be implemented. Another possibility is the region-dependence of the injection over the cerebellar cortex. As suggested by Mitoma et al. (2021), both enhanced GABAergic input, impaired LTD, reduced parallel-fiber synaptic transmission, and multiple innervations of climbing fiber would disrupt the excitation/inhibition balance (i.e., excess inhibitory conductance) of Purkinje cells. The lowered intrinsic excitability of Purkinje cells may disrupt the circuit dynamics among the cerebellar cortex and nuclei, which results in the ataxia generation [44].

## 2. Genetically Induced Cerebellar Ataxia

Acute cerebellar inflammation due to infection is comparable to the cases caused by genetic mutations. Mechanisms for genetically induced cerebellar ataxia were discussed and reviewed well in other studies [44,45,46,47,48]. To understand the mechanism of emergence of the cerebellar ataxia by postinfectious inflammation and the neurophysiological aspects, we would like to initiate the review with the introduction of a couple of ataxia studies, concerning cerebellar ataxia caused by a gene mutation. There are several classifications of genetically induced cerebellar ataxia: autosomal dominant ataxias and autosomal recessive ataxias. Autosomal dominant ataxias are represented by spinocerebellar ataxias (SCA) and episodic ataxias (EA). According to Shakkottai & Fogel (2013), SCAs have 40 classifications with identification of the specific locus of genes, including ATXNs, SPTBN2, CACNA1A, TTBK2, PPP2R2B, KCNC3, PRKCG, ITPR1, TBP, KCND3, PDYN, FGF14, AFG3L2, BEAN1 and TK2, TGM6, NOP56, and ATN1 [49]. Many of these gene products directly and indirectly modulate the intrinsic excitability of neurons, synaptic transmission, and induction of those forms of plasticity. We may note that SCAs are not the only inherited form of cerebellar ataxia, typified by pyramidal signs, amyotrophy, extrapyramidal signs, ophthalmoparesis. In dentatorubral-pallidoluysian atrophy (commonly known as DRPLA), patients show atrophy of the cerebellar dentate nucleus with the symptoms of involuntary movements, mental and emotional problems, and a decline in cognitive ability. DRPLA is caused by a mutation in the ATN1 gene and most common in the Japanese population. Studies on hemiplegic migraine also suggest its relevance to the SCA6-responsible gene CACNA1A, which encodes a subunit of P/Q type voltage-gated calcium channel (Cav2.1) [50]. In EAs, episodic ataxia type 1 and type 2 (EA1 and EA2, respectively) are most commonly identified. Interestingly, CACNA1A is also identified gene/chromosomal location of EA2. The episodes are triggered by stress, startles, and muscle twitching at the onset of childhood, adolescence, and early adulthood [51].

In contrast, autosomal recessive cerebellar ataxia is a large group of degenerative and metabolic disorders. Autosomal recessive ataxias include Friedreich ataxia, ataxia-telangiectasia, congenital cerebellar ataxia, and Wilson’s disease. For instance, GRM1 is identified as the mutated gene of congenital cerebellar ataxia, which encodes metabotropic glutamate receptor mGluR1. mGluR1 is highly expressed in cerebellar Purkinje cells and plays a crucial role in cerebellar development and the plasticity of the neuron [52]. Friedreich ataxia (FRDA) is the most common inherited ataxia and is a human autosomal recessive disease caused by a 90-1300 GAA triplet repeat expansion in the gene encoding the frataxin, a mitochondrial protein involved in iron metabolism [53]. This GAA repeat expansion in the frataxin gene (FXN) is homozygous for 95–98% of patients with FRDA [54,55]. A recent study found a rare compound heterozygous expansion of GAA repeat in one allele, whereas the other allele contains different pathogenic mutations [56]. This dynamic mutation in the FXN gene decreases the amount of frataxin protein and therefore leads to FRDA-like symptoms [57]. Frataxin knockout mice showed motor coordination defects, loss of sensory neurons in dorsal root ganglia and their corresponding axons in nerve roots, a myelin-sheath decompaction enhancing the adaxonal space, and demyelination of axons [58]. This demyelination and neurodegeneration lead to FRDA [59].

According to Rossi et al., almost 100 different genetic entities have been identified in patients with genetically confirmed recessive cerebellar ataxias [45]. Pathophysiological mechanisms underlying autosomal recessive cerebellar ataxias are mediated by the specific vulnerability to the cellular metabolic systems: mitochondrial defect, DNA breakdown, repair dysfunction, RNA transcription or processing defect, altered lipid metabolism, axonal dysfunction, abnormal myelin structure or composition, disrupted intrinsic Purkinje cell firing, synaptic dysfunction, calcium homeostasis dysregulation, lysosomal dysfunction, and disrupted autophagy [46,60]. Generally, such cerebellar ataxia is progressive and worsens in their lives. These behavioral deficits are associated with cerebellar atrophy and loss of Purkinje cells [48]. Reduced Purkinje cell action potential firing has been shown in mouse models of spinocerebellar ataxia and EA2 [61,62,63,64]. In SCA1 model mice, Purkinje cells show lower firing frequency, prolonged mGluR1-dependent synaptic currents by parallel fibers, and calcium signaling, as well as the ataxic phenotype. Administration of a selective mGluR1 antagonist JNJ16259685 ameliorated the ataxia-like behaviors [65]. A selective agonist of GABA_B_ receptor Baclofen also improved the behavioral disruption of SCA1-Tg mice, presumably via the functional crosstalk between G-protein-coupled receptors (GPCRs) [66]. A disruption of the Src family of protein tyrosine kinases (SFKs) signaling and an increased intracellular calcium concentration of Purkinje neurons are suggested to lead to the Purkinje-cell loss and behavioral deficits in those transgenic mice [48,65].

Other studies on the essential tremor pointed out the decreases in the parallel fiber synaptic spines, excessive GABAergic innervation to Purkinje cells from basket cell, and reduction of slow hyperpolarization via large-conductance, calcium-activated potassium (BK) channel [47,67,68,69]. Essential tremor is one of the most prevalent movement disorders. It is considered linking cerebellar neurodegeneration and its dysfunction. In patients with essential tremor, dendritic arborization, and dendritic spines of Purkinje cells are reduced [70], the GABAergic projections from the cerebellar cortex to the dentate nucleus are defective [71,72], and the cerebello-thalamo-cortical network is abnormal [73,74]. According to Hua & Lenz, during the postural tremor, 51% of the neurons in the ventral intermediate thalamus (Vim) exhibited a dense power at a tremor frequency that was correlated with electromyography, and they call them tremor neurons [28]. An in silico study suggests that a progressive loss of GABA_A_ α1-receptor subunits and upregulation of α2/3-receptor subunits in the dentate nucleus closely reproduced experimental evidence (at the peak frequency of Vim within the band 4–12 Hz) forwarding the cerebello-thalamo-cortical network [75]. The study showed that an alteration in the amplitude and decay time of the GABAergic synaptic currents in the dentate nucleus can facilitate the sustained oscillatory activity at the tremor frequency, which is consistent with the observations of thalamic tremor cells in essential tremor patients [28]. Furthermore, the in silico study also shows that tremor frequency could decrease as the high-frequency stimulation, mimicking the deep brain stimulation, is delivered to Vim [75]. Thus, it is argued that the origin of the tremor generation in essential tremor is via a cerebello-thalamo-cortical network [47,75].

In the mouse models, one of the well-studied transgenic mice with an ataxia phenotype is the GluD2 (also known as delta2) mutant mice [76,77,78]. GluD2 molecule is specifically expressed in the cerebellar Purkinje cells, and the molecule was originally shown to play an essential role in the parallel-fiber Purkinje cell’s synaptic transmission and synaptic plasticity: long-term depression (LTD). In GluD2 knockout mice, impaired LTD, reduced parallel-fiber transmission, and multiple innervations of climbing fiber were characterized [76,77,78]. Multiple innervations of climbing fibers are observed in GluD2 knockout mice and are considered as a compensatory mechanism of afferent fiber innervation [77]. The resultant but prominent feature is an increase in the climbing fiber input, compared to wild-type, presumably due to an increase in the error information from the inferior olivary nucleus in vivo [79]. The impairment of parallel-fiber LTD and the loss of learning were assumed to increase error signals from climbing fibers [79]. The increase in the activity of climbing fibers (an afferent glutamatergic excitatory fiber from the inferior olivary nucleus to the cerebellar Purkinje cell) promotes the induction of the enhancement of GABAergic synaptic transmission via the induction of the long-term potentiation [80]. The increase in GABAergic transmission implied suppressing the over-excitability of GluD2-deficient Purkinje cells by the excess excitatory drives and to maintain cell homeostasis, although without fine-tuning. The phenotype of the increase in the inhibitory GABAergic synaptic transmission is also observed in the plasma membrane Ca^2+^-ATPase isoform 2 (PMCA2) knockout mice, which show severe ataxia [67]. Associated with GluD2, an old mutant mouse Lurcher is characterized by ataxia [81]. Lurcher mouse is a spontaneously occurring autosomal dominant mutation that caused the degeneration of virtually all cerebellar Purkinje cells [82]. Lurcher is a gain of function mutation in the GluD2 that converts the receptor into a constitutively leaky cation channel [83]. In the heterozygous Lurcher mutant (+/Lc), Purkinje cells die due to excitotoxicity, while, in turn, olivary neurons and granule cells degenerate due to the loss of their Purkinje cell targets [84]. Tippy mutant mouse is another example of a spontaneous neurological mouse mutant with cerebellar atrophy, reduced parallel fiber synapses, and ataxia [85]. There are other cerebellar mutant mice classically known with ataxia, such as Hotfoot mice, Purkinje cell degeneration mice, Nervous mice, Staggerer mice, Weaver mouse, Reeler mouse, and Scrambler mouse [53]. However, those phenotypes have already been reviewed in another excellent study [53]. Thus, we would like to avoid further explanation. Together, in the cerebellar cortex, the dysfunction and impairment of parallel fiber synaptic transmission and plasticity, spine and dendrite abnormalities, innervation from afferent fibers, and the enhancement of GABAergic transmission, as well as the hypoexcitability and neurodegeneration of Purkinje cells, are manifested in the many animal models with the ataxic phenotype.

In the following sections, we discuss the cerebellar ataxia associated with infection and aberrant immune stress (Figure 1). First, we introduce the infectious disease models by microorganisms and the resultant cerebellar atrophy and neurodegeneration. Next, we discuss the possibility of the disruption of the brain vasculature system. Lastly, we discuss the neurophysiological modulation, microglia-related dysfunction of the cerebellum-involved circuit, and ataxia genesis.

## 3. Cerebellar Inflammation via Infectious Agents and Host Immune Response

### 3.1. Viral Infections

Viral pathogens are the most common causative agents of inducing para-infectious, post-infectious, or post-vaccination cerebellar inflammation. Among the viruses, Epstein-Barr virus (EBV), rotavirus, herpes simplex virus, dengue virus, varicella-zoster virus, parvovirus B19, enterovirus, echovirus, West Nile virus, coxsackievirus, influenza virus (Influenza A and B), respiratory syncytial virus, human herpesvirus, mumps virus, and adenovirus have been reported [86]. The involvement of herpes simplex virus type 1 (HSV-1) in acute cerebellitis is rare and was reported in only two different studies with three cases. In one case, the patient was previously diagnosed with human immunodeficiency viruses (HIV)-1 infection. In the other two cases, the patient did not have any immunodeficiency [87]. The infections of HIV caused the primary cerebellar degeneration and related to other opportunistic pathogens, such as the EBV in acute cerebellitis [88].

#### 3.1.1. Cytomegalovirus

Acute cerebellitis in adults is very rare and only 35 cases have been reported between 1991 and 2017, where most cases were female (63%). The etiology of viral pathogens was revealed in 22% of cases, while in 34% of the patients, pathogens were not screened [89]. According to Samkar et al., either EBV or cytomegalovirus (CMV) was the possible reason in the cases of acute cerebellitis in adults because of the presence of both EBV IgM and CMV IgM in cerebrospinal fluid (CSF); however, the study was unable to isolate the pathogens themselves. They suggested it as a para-infectious phenomenon and doubted about other pathogens or auto-immune as the cause [89].

CMV is one of the leading causes of developmental brain damage which induces cerebellar inflammation upon infections in the fetus, exhibiting deficits in cerebellar cortical development. Kosmac et al. investigated cerebellar inflammation in murine cytomegalovirus (MCMV)-infected mice [90]. In response to viral infection, activated immune cells, such as mononuclear cell, Iba-1 expressing microglia, and/or macrophages, were shown to infiltrate from the periphery and to produce inflammatory cytokines: tumor necrosis factor (TNF)-α, interferon (IFN)-β, signal transducer and activator of transcription 1 (STAT1), and an IFN-induced protein with tetratricopeptide repeats 1 (IFIT1) to exacerbate inflammation in the cerebella [90]. A related study showed that the morphological abnormalities of the cerebellar cortex included increased thickness of the EGL, decreased thickness of the IGL, abnormal arborization of Purkinje neuron dendrites, and thinning of the molecular layer [91]. The expression of chemokines, MCP-1 (Monocyte chemotactic protein 1, also known as C-C motif chemokine 2 (CCL2)) and RANTES (also known as CCL5) is also increased over the course of acute infection, which may promote the infiltration of immune cells into the cerebella [92]. MCP-1 and RANTES were previously reported requisite for the infiltration of myeloid and lymphoid cells into the central nervous system (CNS) following MCMV infection [93].

#### 3.1.2. Influenza Virus

Recently, a 2-year-old boy had been reported with acute cerebellitis after being diagnosed with the influenza A virus [94]. While several cases with acute cerebellitis by influenza virus were reported, most cases were in children and adult females [95]. The infection with the influenza virus causes acute inflammation via a direct invasion into the CNS, resulting in the excessive production and action of proinflammatory cytokines including TNF-α and interleukin (IL)-6. An excess release of the proinflammatory cytokines causes vascular endothelial injury and apoptosis of parenchymal cells, which further results in brain edema and systematic organ damage [96]. Blackmore et al. found that the infection increased the level of circulating IL-6, though without any changes in the levels of IL-1β, TNF, IL-17A, and IL-10 [97]. They investigated it in an autoimmune-prone T-cell receptor transgenic mouse (2D2) inoculated with the influenza A virus. The authors showed that the viral infection increased T-cells in the choroid plexus and induced a temporal transcriptomic alteration in the cerebellum. The upregulated genes by the inoculation and the induction via INF signaling lead to activation of glial cells [97]. In influenza-inoculated mice, several chemokines, including CCL6, CCL17, CCL25, CCL28, and C-X-C motif chemokine 5 (CXCL5), increased, while CCL27a and CX3CL1 decreased. These data provide evidence of an increase in immune-cell surveillance in the cerebellum followed by influenza infections [97].

#### 3.1.3. Dengue Virus

Dengue cerebellitis is also rare, which was first reported by Karunarathne et al. (2012), while it was caused by a co-infection with EBV [98]. In the following year, Weeratunga et al. reported three recent cases of cerebellitis associated with only dengue fever [99]. As of 2021, a total number of 10 cases has been reported for cerebellitis associated with dengue fever, and these cases were in adults [99,100,101,102,103,104,105]. The pathophysiology of dengue cerebellitis is yet to be discovered. It is postulated that this cerebellitis may result from the direct invasion of the virus and immune-mediated mechanism [104]. The hematogenous route is considered the most probable entry of Dengue virus (DENV) into the CNS [106]. To elucidate the mechanism of DENV infection in the brain and cerebellum, an investigation was done in BALB/c mice inoculated by DENV 2 strain [107]. This study showed the replication of the dengue virus in the cerebral tissue, as well as the morphological alteration of the microglia and astrocytes in the nervous tissue of the infected mice. Dengue infection resulted in the activation of microglia and the drainage of antigens, after a milder inflammatory reaction in the cerebellum [107]. Activated T cells also migrate into the cerebellum with higher expression of adhesion molecules, which are stimulated by pro-inflammatory cytokines. Cytokine and chemokine levels increase in brain tissue from DENV-3 infected mice. The infection elicits significant increases in IFN-γ, TNF-α, CCL2, CCL5, CXCL1, and CXCL2 [108]. Additionally, astrocytes are activated together with microglia, and they produced pro- or anti-inflammatory cytokines and chemokines for influencing the T-cell responses [107,109]. The production of inflammatory mediators, such as TNF-α, IL-6, IFN-γ, granulocyte-macrophage colony-stimulating factor (GM-CSF), and IL-1, possibly promotes the breakdown of the blood–brain barrier’s (BBB) integrity; increases the permeability; leads to the infiltration of mediators, immune cells, and microorganisms; and stimulates the differentiation and proliferation of astrocytes and microglial cells in parenchyma [110].

#### 3.1.4. SARS-CoV-2

The world is currently going through a global pandemic of severe acute respiratory syndrome coronavirus 2 of the genus Betacoronavirus (SARS-CoV-2), which causes coronavirus disease: COVID-19. The main reason for the COVID-19 outbreak is a rapid person-to-person transmission of SARS-CoV-2 [111]. The frequent mutations that occurred in this virus also make it more challenging to develop therapeutics [112]. Even those having organized health systems, every country is facing difficulties to end COVID-19 [113]. The newly emerged Omicron variant of SARS-CoV-2 can infect vaccinated people and thus has increased the risk of SARS-CoV-2 reinfection [114]. As of 8 February 2022, this virus has already infected approximately 400 million people worldwide [115]. A few cases of acute cerebellitis associated with SARS-CoV-2 were reported, though SARS-CoV-2 itself was rarely detected in CSF [116]. The first case was presented by Fadakar et al. in a 47-year-old man. The authors confirmed the virus in CSF through PCR. Direct invasion of the virus into the nervous system may be the probable pathogenic mechanism in their patients [117]. Another case study of COVID-19-related acute cerebellitis suggested an increase in the protein level and a lymphocytosis in CSF [118]. The number of cases with acute cerebellitis and encephalitis associated with SARS-CoV-2 is increasing, though the pathogenic mechanism is still unclear. In addition to the direct viral invasion, the cytokine storm is presumed as the possible mechanism for acute cerebellitis by SARS-CoV-2 [119]. Infection with SARS-CoV-2 drives the release of numerous pro-inflammatory cytokines, which can directly pass BBB and can activate microglia and astrocytes [120,121]. Meanwhile, a recent study has shown the inflammatory responses, with vasculitis, glial activation, and upregulation of inflammatory mediators following SARS-CoV-2 infection [119]. The SARS-CoV-2 is able to traverse BBB in a transcellular pathway by disrupting the basement membrane. Indeed, upon SARS-CoV-2 infection, the expression of MMP9 increases, while the expression of collagen IV decreases [122]. Additionally, the spike protein of SARS-CoV-2 disrupts the BBB integrity via RhoA activation [123]. SARS-CoV-2 was suggested to damage the choroid plexus epithelium, which led to the leakage of the blood-CSF barrier in human brain organoids [124]. Thus, infections to viruses induce the inflammatory responses by releasing inflammatory mediators in the brain and cerebellum, damping the vasculature barriers.

COVID-19 infection caused by SARS-CoV-2 is now considered a risk factor of demyelination of the CNS, as well as the peripheral nervous system [125]. Several cases have already been presented for acute demyelination after COVID-19 infection [126,127,128,129]. Recently, a study described acute demyelination of CNS after COVID-19 vaccination in a case diagnosed with hereditary cerebellar ataxia previously [130]. Additionally, SARS-CoV-2 non-structural protein 3 (nsp3) contains a SARS-unique domain (SUD), which is divided into three parts, including the N-terminal, Middle, and C-terminal parts, named as SUD-N, SUD-M, and frataxin-like domain (SUD-C), respectively. This SUD domain can interact with G-quadruplexes (G4s) [131]. G4s perform multiple functions providing a link between G4s and many diseases, including neurological diseases. Abnormal repeat sequence expansion in neurological genes causes the formation of G4s, inducing DNA–RNA hybrids, known as R-loops [132]. GAA repeat expansion in the FXN gene also forms R-loops in patients with FRDA. The increased level in the R-loops causes transcriptional silencing of the FXN gene and triggers the onset of FRDA [133]. Now, it is an open question whether the interaction between SUD and G4s has any role on the formation of R-loops as well as on the onset of the neurological disorder including ataxia.

### 3.2. Bacterial Infections

Cerebellar inflammation associated with bacteria is very rare, too. Accumulating evidence has suggested that blood-borne bacteria can traverse the BBB and induce brain inflammation, which disturbs the functions of neurons and glial cells [134]. The bacteria involved in meningoencephalitis can produce cerebellar signs and symptoms [135]. Both Gram-positive and Gram-negative bacteria contain peptidoglycan (PGN) as their cell wall component. PGN may contribute to the inflammatory processes acting as a stimulant and may promote the infiltration of macrophages and neutrophils in the brain parenchyma from the peripheral [136]. However, the transient exposure to PGN does not change the neuronal activity in cerebellar neurons [30]. LPS, an endotoxin, is the outer component of Gram-negative bacteria and activates innate inflammation via the patter-recognition receptors (i.e., Toll-like receptors (TLRs)), which drive the invasion into the brain [29,30,31]. Activation of TLR4 on the immune-cell surface results in the activation of hundreds of inflammatory transcriptomes, including pro-inflammatory cytokines such as TNF-α, IL-6, and pro-IL-1β [137]. It is noteworthy that all the inflammatory responses are not always via the translation of genes. Macrophages secrete TNF-α rapidly upon their activation through the non-constitutive pathway [138], which triggers the plasticity of neurons without gene expression [30,31]. TNF-α, IL-1β, and IL-6 are induced and amplified by microglia following the stimulation by LPS, while c-Jun N-terminal kinase (JNK)/p38, external signal-regulated kinase (ERK)/JNK, and ERK/JNK/p38 are involved in their induction, respectively. According to Ishijima and Nakajima (2021), microglia may use specific combinations of MAPKs to induce different inflammatory cytokines [139]. Therefore, we may consider that there are two phases of neuroinflammatory responses of non-conventional and conventional pathways. These modulations in physiological properties of neurons may be different between the very early phases of infections, which may exhibit the differences in the symptoms.

### 3.3. Other Infections

Fungal infections, particularly *Aspergillus* infections, cause acute cerebellitis through their invasion into the brain [135]. There are a few cases of cerebellar infections associated with *Aspergillus* [140]. *Aspergillus* spreads to CNS with a hematogenous spread directly or as a primary intracranial lesion [141]. Notably, fungal infections are increasing at an alarming rate because of the growing number of immunocompromised patients, the widespread use of immunosuppressive drugs, and the spread of infectious diseases such as AIDS and COVID-19 [142,143]. These fungal infections are secondary to other infections elsewhere in the body and spread to the brain through blood circulation [142].

Malaria can cause cerebellar impairment during the acute stage of fever as a sequela of cerebral malaria in survivors. The side effects of anti-malarial drugs may cause this cerebellar impairment, even after the successful treatment [144]. However, we would like to note that cerebellar signs are diagnosed in post-malaria neurological syndrome (PMNS), with an inefficacy of anti-malarial drugs in 30% of overall PMNS cases [145].

### 3.4. Potential Involvement of mtDNA in Infection

Mitochondrial DNA (mtDNA) released in infection is potentially involved in cerebellar dysfunction. Dominant roles of mitochondria are metabolism, calcium homeostasis, and cell death. Most ATP in mammals is produced in mitochondria through glycolysis and the citric acid cycle (i.e., TCA cycle (tricarboxylic acid cycle) or the Krebs cycle). The driven electron transport chain converts ADP to ATP by oxidative phosphorylation in the folds or cristae of the inner membrane. Infection with human pathogen HSV-1 induces rapid and complete degradation of host mitochondrial DNA during the infection of cultured mammalian cells, in which viral UL12.5 isoform gene localizes to mitochondria and triggers mtDNA depletion [146,147]. It is remarkable that the injection of mtDNA was shown to induce inflammatory reactions on arthritis development in mice, presumably via monocytes/macrophages [148]. In contrast, nuclear DNA does not induce inflammation because of containing methylated CpG sequences [148]. TLR9 selectively recognizes unmethylated CpG dinucleotides in bacterial DNA as the ligand. Therefore, the mtDNA-TLR9 pathway would be responsible for the inflammation. According to Riley & Tait, mtDNA can trigger various pro-inflammatory signaling pathways by endosomal localized TLR9 or via cytosolic cGAS-STING or via cytosolic inflammasome (AIM2 or NLRP3) [149]. Both mtDNA and mitochondrial double-strand RNA activate TLR9 and TLR3, respectively. Upon infection with HSV-1, cytosolic mtDNA stimulates the production of type I IFN via the RNA polymerase pathway [150], driving the inflammation. Infection with the dengue RNA virus is also shown to activate human dendritic cells via TLR9 signaling [151]. Therefore, mitochondrial dysfunction upon infection in the cerebellum could be involved in the emergence of acute inflammatory ataxia. Indeed, the repair of mtDNA damage extends the lifespan of mutant ataxin-1 knock-in mice of an SCA1 model [152]. In hypoxia situations, microglia activation was shown to induce LTD of synaptic transmission and activity of hippocampal CA1 pyramidal neurons via reactive oxygen species (ROS)-involved signal pathway [34]. This would be another pathway of the potential involvement of mitochondria for the neural dysfunction in acute inflammation in the brain, while this field is not yet well investigated.

### 3.5. Cerebellar Atrophy and Host Autoimmunity

The relevance of cerebellar atrophy to cerebellitis is observed in a case study with two patients [153]. Those two patients showed isolated cerebellar atrophy in late-stage cerebellitis [153]. Pro-inflammatory cytokines, including IL-6, IL-9, IL-12, IL-13, GM-CSF, and macrophage inflammatory proteins-1α (MIP-1α/CCL3), increased in patients with cerebellar atrophy and induced inflammation in the cerebellum. MCP-1 negatively correlated with cerebellar atrophy patients, whereas CSF MCP-1 levels were higher at the early stage and gradually decreased over time (e.g., more than several months) [154]. Note that the early stage of human patients is often already in the chronic phase in rodent models, which could mislead our interpretations. MCP-1 causes massive infiltration of macrophages in the early phase of cerebellar atrophy [155].

Additionally, a recent case study described the immune-mediated cerebellitis that had developed after the treatment of primary refractory Hodgkin lymphoma with immune checkpoint inhibitors [156]. Cerebellar autoimmunity is known to be triggered in patients with gluten ataxia, postinfectious cerebellitis, Miller Fisher syndrome/Guillain-Barre syndrome, opsoclonus-myoclonus syndrome, and paraneoplastic degeneration [44]. Autoantibodies relate to cerebellitis. Cerebellar Purkinje cells highly express a scaffold molecule Homer-3, and its antibodies also induce cerebellitis and ataxia [157,158]. Furthermore, antibodies against glutamic acid decarboxylase (GAD), GluD2, Caspr2, mGluR2, AP3B2, ITPR1, and NIF are also suggested to link with immune-mediated ataxia, although the mechanism remains poorly understood [159,160,161,162]. Several environmental factors, including exposure to toxic chemicals, gluten-containing diets, and diseases, such as cancer, affect the formation of autoantibodies [163]. In addition, cell-mediated autoimmunity is considered immune-mediated cerebellar ataxia (IMCA). The number of CD8+ T cells is increased in CSF in some subtypes of IMCAs. CD8+ T cells may infiltrate into the cerebellum along with the macrophages. Therefore, CD8+ T cells are suggested to play a pathogenic role in the IMCAs [163]. While we cannot conclude that identical cellular mechanisms are involved in auto-immune diseases and in infectious diseases, both of which disrupt cerebellar function and emerge cerebellar ataxia, these studies would supplement our interpretation of the disorder.

GAD is involved in the synthesis of inhibitory neurotransmitter GABA with a rate-limiting step [164]. There are two major types of GAD: GAD65 and GAD67. GAD65 synthesizes GABA for neurotransmission and synaptogenesis, whereas GABA produced by GAD67 is considered to not be related to neurotransmission and synaptogenesis [165]. GAD antibodies (GAD Abs) disturb the cerebellar activity by the suppression of GABA release, which leads to cerebellar ataxia. Thus, GAD Abs alter the balance between glutamate and GABA and induce glutamate excitotoxicity [166,167]. GAD65 Abs play a major pathogenic role in clinical manifestations of cerebellar ataxia, leading to the development of cerebellar ataxia [168]. GAD65 Abs cause neurological impairments in an epitope-specific fashion that elucidates the diversity of neurological represents in patients with GAD65 Abs [169].

Gluten is a dietary protein and often triggers autoimmunity in the cerebellum. Gluten antibodies cross-react with cerebellum tissue and cause immunological damage to the cerebellum, leading to the ataxia, termed gluten ataxia [170]. This cross-reactivity occurs between antigenic epitopes on Purkinje cells or other cerebellar cells (granular layer) proteins and gluten peptides [171]. There is a surprising association between anti-GAD ataxia and gluten ataxia. Gluten sensitivity is a part of the underlying pathogenesis of anti-GAD ataxia [172]. Hadjivassiliou et al. showed that the gluten-free diet reduced the titer of anti-GAD antibodies, suggesting a link between neurological manifestations of gluten sensitivity and anti-GAD ataxia [170]. While transglutaminase 2 (TG2) plays a significant role in the pathogenesis of gluten ataxia [173], TG2 deamidates gluten peptides and increases their reactivity with HLA DQ2/DQ8, resulting in the stimulation of the T cell response. Both TG2 activation and gluten peptide deamidation are considered crucial for the development of gluten ataxia [174].

Opsoclonus-myoclonus syndrome (OMS) is a rare neurological disorder with clinical features of opsoclonus, myoclonus, and ataxia [175]. Opsoclonus is an abnormal nystagmus that causes agile and unordered eye movements. Myoclonus is involuntary muscular contraction around the head and shoulders. OMS is a rare idiopathic or paraneoplastic syndrome, which is mainly associated with breast carcinoma, lung cancer, and ovarian teratoma [176]. OMS patients have neuronal surface antibodies including glycine receptor antibodies (GlyR Abs), which can interact with the L2 epitope of glycoproteins in the CNS [177]. GlyR Abs are suggested to disturb the inhibitory (i.e., glycinergic) circuits [178]. Nevertheless, the exact mechanism remains elusive.

Genetic alterations or amplification of genes in tumor cells induce the breakdown of immune tolerance and initiate the auto-immunity in paraneoplastic cerebellar degenerations with anti-Yo antibodies (Yo-PCD). Most mutations with gain of function occur in CDR2 (Cerebellar Degeneration-related Antigen-2 (62kD)/Yo Paraneoplastic Antigen) and/or CDR2L (Cerebellar degeneration-related protein 2-like) genes. However, the mechanism differs from patient to patient where individual or combination of abnormalities in CDR2 and/or CDR2L may not be the same in all patients [179]. The association of HLA with Yo-PCD was observed in many cases. The frequency of the HLA-DRB1*13:01–HLA-DQA1*01:03–HLA-DQB1*06:03 MHC class II haplotype was increased in Yo-PCD ovarian cases [180]. However, patients with Hu-antibody-associated paraneoplastic neurological syndromes (Hu-PNS) showed a higher frequency of both HLA-DR3 and HLA-DQ2 alleles, supporting the role of CD4+ T cells in its pathogenesis [181]. The uptake of anti-neuronal antibodies by neurons suggests a direct neurotoxic role of autoantibodies. CDR2 and CDR2L antibodies increase the expression of voltage-gated calcium channel Cav2.1, protein kinase C gamma (PKCγ), calcium-dependent protease, and calpain-2 after their independent internalization by Purkinje cells. This internalization of ion channels hampers the calcium homeostasis of Purkinje cells, resulting in neural dysfunctions and loss of neurons [182].

## 4. Possibility of the Inflammatory Cytokine Infiltration and Disruption of the Vasculature Barriers

The BBB physically separates the CNS from the peripheral blood circulation and actively regulates the influx and efflux of solutes, molecules, cells, and pathogens [183]. It protects the brain from toxins and pathogens and allows neurons to function properly by maintaining a tightly regulated milieu [184]. At the early stage of neuroinflammatory diseases, BBB disruption could occur through intrinsic and/or extrinsic effects. The BBB limits the entry of most pathogens by its strong cell–cell adhesion, composed of adhesion molecules of tight junction: claudins, occludin, and junctional adhesion molecule (JAM). Nevertheless, neurotropic viruses, molds, and certain parasites are able to cross the barrier and infect CNS parenchyma. These pathogens lead to robust inflammatory responses and extensive neutrophilic infiltrates, resulting in BBB disruption [185]. Other factors, such as pro-inflammatory cytokines and chemokines, including IL-1β, IL-17, IL-22, TNF-α, IFN-γ, and CCL2, are released by leukocytes during transmigration, which loses the integrity of the BBB. IL-1β induces the expression of matrix metalloproteinase-9 (MMP-9) and indirectly destabilizes the BBB [186].

## 5. Neurophysiological Modulation of Intrinsic Excitability

Ion channels play a crucial role in neurophysiological modulation: intrinsic excitability and excitatory and inhibitory synaptic transmission. In this review, we would like to focus on the forms of plasticity of neuronal intrinsic excitability. As introduced, ion channels are directly linked to the neural dysfunctions in the cerebellar ataxia. Voltage-gated and ligand-gated channels are two major categories, where the former induces ionic currents on the cell surface and determines the membrane property of neurons, and the latter locates in the postsynaptic membrane and generates synaptic conductance [187]. The voltage-gated potassium-, calcium-, and sodium- channels are mostly involved in the generation, formation, and regulation of the action potential of Purkinje neurons in the cerebellum [188].

Both BK and SK channels are expressed in the Purkinje cells. Depolarization and intracellular Ca^2+^ activate these channels in Purkinje cells. BK channels are localized in the paranodal junction and generate slow afterhyperpolarization [189,190]. There are two types of voltage-dependent responses observed: a passive Na^+^ spiking with a following plateau-like depolarization associated with a prominent conductance change that further produces large dendritic action potentials as observed in the complex spike [191]. These potentials elicit burst firing of the Purkinje cells [192,193]. BK and SK channels are not necessarily required for regular spontaneous firing, while P/Q-type calcium channels are essential for regulating burst firing in Purkinje neurons. Blocking of P/Q-type calcium channels disturbs the generation of dendritic calcium spikes and results in irregular bursts. SK and BK channels are involved in interspike and interburst intervals [194]. SK channels are Ca^2+^- and voltage-dependent, whereas BK channels have two distinct conductances, including inactivating Iberiotoxin (IbTX)-sensitive and non-inactivating IbTX-insensitive conductance. IbTX-sensitive current activates rapidly and contributes to spike repolarization, whereas slowly activating IbTX-insensitive current regulates the interspike interval [195]. A study using the BK-channel-deficit mice demonstrated the loss of eye-blink conditioning and cerebellar dysfunction. Lack of BK channels causes their motor discoordination and ataxia [77].

SK channels are classified into SK1 (KCa2.1), SK2 (KCa2.2), and SK3 (KCa2.3). The blockade of SK channels develops cerebellar ataxia, as mentioned previously. SK channels have an important role in regulating the firing frequency of neurons in both the cerebellar cortex and deep cerebellar nuclei (DCN). A transgenic mouse model expressing SK3-1B-GFP, which dominant-negatively suppresses SK channel function, shows the ataxia phenotype [196]. Transgene expression was restricted to the DCN within the cerebellum due to the Thy1.2 promoter. In that model, cerebellar ataxia was detectable at postnatal day 10, concomitant with the onset of SK3-1B-GFP expression [196]. Stimulation of microglia by LPS-exposure in the cerebellar cortex and the resultant hyperexcitability of Purkinje cells were not sufficient to induce cerebellar ataxia, although the injection of 200 nM apamin to the cerebellar anterior lobes induced ataxia [30]. However, bulk injection of apamin could not specify the effect. In contrast, using Purkinje cell-specific promoter L7, SK2-deletion mice have neither major impairment in locomotion nor show ataxia-like tremors, while they display an abnormal locomotion pattern that results in taking significantly longer steps [197]. Together, we agree that the hyperexcitability of the DCN directs the cerebellar ataxia, as previous neurodegenerative disease models have suggested. In addition, SK channels modulate the compartment-specific dendritic plasticity in the cerebellar Purkinje cells, where the downregulation of SK channels is indispensable for the induction of dendritic plasticity [42,198,199]. SK channels have a heterogeneous expression pattern, which may be responsible for the functional clustering on PC branch fields. Moreover, SK channels alleviate the local excitability in the dendritic branch and limit the conductivity of synaptic current in dendrites [199]. Another insight comes from dendritic excitability. Because of the difficulty, usual research of the cerebellar Purkinje cell using electrophysiological profiling is conducted merely by the recording from the neuronal soma. A study of the whole cell-patch clamp from Purkinje cell dendrites indicated that the intrinsic excitability of the dendrites influences the amplitude of the somatically recorded EPSC. Increased excitability of the Purkinje-cell dendrite promotes the conduction of synaptic input from the dendritic branch to the soma [199], providing a new functional aspect of dendrites and a possibility of the excitability modulation of the neuronal dendrites by immunity. Vice versa, the lowered excitability of Purkinje cells could result in a decrease in the amplitude of EPSC. Those could be the cellular modulation of cerebellar ataxia models.

Another voltage-gated channel, the sodium channel Nav1.6 is also associated with cerebellar ataxia. SCN8A gene encodes Nav1.6 protein. The expression of Nav1.6 is regulated by the fibroblast growth factor 14 (FGF14) [200], which is mainly expressed in the CNS neurons and is associated with spinocerebellar ataxia (SAC27). The loss of FGF14 reduces the expression of Nav1.6, which underlies the high-frequency characteristics in Purkinje-cell action potential firing. The dysfunction of Nav1.6 disrupts action potential generation in Purkinje cells, compared to the granule cells. The reduced expression of Nav1.6 might be a pathogenic mechanism of the cerebellar ataxia [200,201,202], while this disease is not necessarily related to aberrant immunity. Recovery of the Na^+^ channels from inactivation is associated with a sizeable ionic current. There are two mechanisms for the recovery of the channels. In the first mechanism, the channels recover without current conductance, while the other mechanism, which is favored by large depolarization, recovers channels passing transiently through the open state. Recovery of these channels promotes the high-frequency firing of Purkinje neurons after the depolarization of cells resulting from the sodium current flowing and changing in the action potential during the recovery process [203].

## 6. The Cerebellar Circuit Dysfunction

The cerebellum contains most of all the neurons in the brain (i.e., granule cells), and these neurons form many of the circuits that control motor coordination and motor learning [204,205]. Glial cells of the cerebellum modulate the synaptic efficacy between neurons and the activity of the neural circuit. The disruption of this glia–neuro interaction can modulate the circuit activity [206]. Microglia, the resident immune cells of the brain, are involved in synapse formation, neural circuit regulation, and plasticity induction. Microglia modulate synaptic activity [207], and they were shown to release superoxide to elongate their processes by which the damaged Purkinje-cell bodies are engulfed [208]. Microglia are reported to remove synapses from damaged neurons in the adult brain [207]. In the developmental cerebellum, microglia permit the elimination of surplus climbing fibers [209]. Remodeling of synaptic connection occurs constantly based on experience and results in synaptic plasticity [210]. Disturbance of synaptic connectivity and neuronal excitability would lead to impaired animal behaviors and promote neurodegenerative diseases [30,211].

After the infection with human coronavirus (HCoV-OC43) in mice, activated microglia and excessive proinflammatory cytokines release cause the downregulation of glial glutamate transporter GLT-1 expression [212,213]. The downregulation of GLT-1 expression leads to the disruption of glutamate homeostasis and influences excitotoxicity [213]. Activated microglia also promote the synthesis of neurotoxic metabolites instead of serotonin, which induces excitotoxicity in neurons [214]. Glutamate excitotoxicity causes excessive Ca^2+^ influx, disruption of Ca^2+^ homeostasis, and increased production of ROS [215]. An excessive Ca^2+^ intake in the postsynaptic neuron induces extreme neuronal firing and hyperactivation of calpain. All these abnormalities further result in progressive motor neuron loss and the pathogenesis of neurodegenerative diseases [216,217].

The release of dopamine is also significantly affected by the increased inflammatory cytokines. Increased INF-α decreases striatal dopamine release, resulting in neural connectivity disruption and psychomotor slowing [218,219]. However, IL-1β plays a crucial role in regulating axonal dynamics and neural circuit activity during acute inflammation. IL-1β contributes to the increased axonal dynamics, the enhancement of branch addition rates, branch loss, and the increased number of transient branches [220].

## 7. Emergence of the Cerebellar Ataxia and Possible Treatment for Acute Cerebellar Inflammation

**Figure 2:**
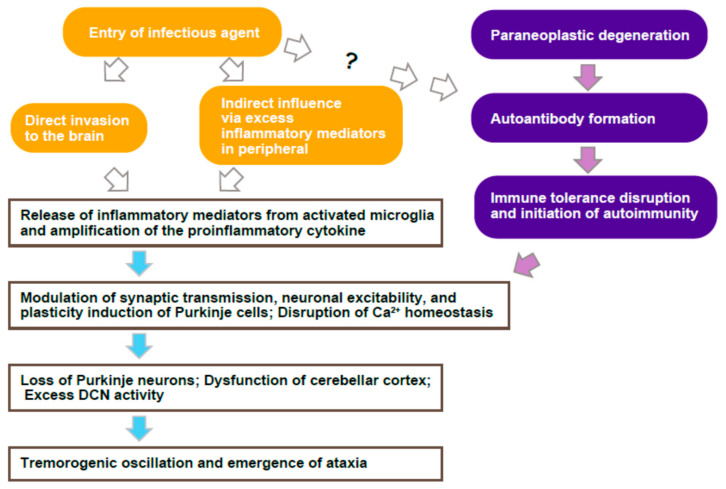
Schema of Cerebellar Ataxia by Infection and Autoimmunity. Infectious agents induce cerebellar inflammation through their direct invasion to the brain or indirectly via influencing excessive inflammatory mediators in the peripheral, resulting in the activation of microglia and the release of excessive inflammatory cytokines. Activated microglia and excessive inflammatory mediators cause neurophysiological modulation of synaptic transmission, plasticity induction of Purkinje cells, and disruption of Ca^2+^ homeostasis, leading to the loss of Purkinje neurons, dysfunction of the cerebellar cortex, and excess DCN activity, followed by tremorogenic oscillation and emergence of ataxia. The formation of autoantibodies is triggered by infectious agents and paraneoplastic degeneration. Although the exact mechanism of autoantibody formation by infection is unclear, autoantibodies disturb the immune tolerance and induce autoimmunity in the cerebellum, resulting in the neurophysiological modulation, plasticity, and disruption of Ca^2+^ homeostasis, merging to the ataxia-generating pathway.

How does cerebellar ataxia emerge after and during cerebellitis and acute cerebellar inflammation? We propose a hypothesized procedure (Figure 2):

1. Infection with pathogens and peripheral inflammation causes the initial release of TNF-α via the conventional and non-conventional pathways in the macrophages and microglia.
2. Released TNF-α amplifies the other proinflammatory cytokine release via gene translation, which possibly elicits the breakdown of the BBB’s integrity, increases the permeability, and promotes the infiltration of mediators, immune cells, and microorganisms.
3. Early inflammatory cytokines (e.g., TNF-α, IL-1β, and IL-6) could modulate synaptic transmission, neuronal excitability, and plasticity induction. In the cerebellum, Purkinje cells induce the forms of plasticity as the increase in the firing frequency, dendritic excitability, the presynaptic release, and postsynaptic responsibility. All the forms of plasticity could result in the deficit of cerebellar involuntary movement and higher cognition by impairing the compensation of the error signals and augmenting the tremor-generating oscillatory activity.
4. After neurodegeneration by excess immunity and activated microglia, Purkinje cells lose their numbers. Prolonged hyperexcitability and cytotoxicity promote neuron loss. The remaining Purkinje cells also show hypoexcitability, a reduction in the parallel fiber synaptic transmission, receive aberrant climbing fiber input, excess GABAergic input, and disrupted output from the cerebellar cortex. The loss of inhibitory input from Purkinje neurons, which frequently degenerate in cerebellar ataxia, may initiate hyperexcitability of DCN. Homeostasis imbalance may occur in the cerebellar cortex.
5. Disruption of the network activity of cerebellum-including circuits (e.g., cerebello-thalamo-cortical pathway) and generation of the oscillatory tremorogenic activity, which drives the emergence of ataxia/tremor symptoms.

Microglia are the major immune cells in the brain and thus play indispensable roles in mediating inflammation in response to brain injury and exposure to infectious agents as an innate immune response [221,222,223,224]. Activated microglia secrete a wide range of proinflammatory mediators, including TNF-α, IL-1β, IL-6, chemokines, excitatory amino acids, and ROS [225]. It is speculated that the inhibition of microglia activity could be a potential therapeutic target for acute cerebellar inflammation and ataxia [226].

Rifampicin and trans-cinnamaldehyde (TCA) inhibit microglial activity by blocking the NF-kB signaling pathway. These compounds also suppress the release of proinflammatory mediators [226,227]. After the activation of microglia, the infiltration of T-lymphocytes and macrophages into the cerebellum can be prevented by an antibiotic Rifaximin. Thus, the reagent could impose another therapeutic option for treating cerebellar inflammation. They also suggest that Rifampicin restored motor incoordination by peripheral inflammation in rats [228]. It is interesting that a tropical Southeast Asian fruit named noni (*Morinda citrifolia* L.) showed anti-inflammatory activity in microglial cells. Noni decreased the microglia-mediated inflammation by downregulating the expression of NF-κB; therefore, noni can be used against inflammation by hyperactive microglia [229]. Oxytocin, a peptide hormone produced in the hypothalamus, may attenuate the overactivation of microglia. This hormone reduces the expression of pro-inflammatory mediators and cytokines by inactivating the MAPK signaling pathway [230].

Potassium channels play a key role in the activation of microglia. The blockade of these channels is reported to inhibit microglial activation [231]. 4-aminopyridine (4-AP) is the most promising potassium channels blocker, which likely targets the voltage-sensitive Kv1 (also known as Shaker and KCNA) family. However, at higher concentrations, 4-AP can block a large array of potassium channels [71,232]. 4-AP treatment may cause the modification of the activity of Purkinje neurons and induce serious side effects [188,233]. In contrast to 4-AP, BDS-I slows the activation of the channels rather than completely blocking it. This mode of action could be beneficial for the border pharmaceutical context as it does not affect the Purkinje neurons’ activity and excitability. Small molecules, having a similar modes of action to that of BDS-I, could be developed to limit the activity of potassium channels, as well as the activation of microglia [233].

## 8. Conclusions

This review described the acute cerebellar inflammation and emergence of ataxia from different perspectives. The inflammation in the cerebellum occurs during and after infections or due to the host autoimmunity. Here, we discussed a series of studies that reported acute cerebellitis associated with infectious agents, including viruses, bacteria, or fungi. Immune-deficient individuals are prone to develop cerebellar inflammation upon viral infections, while cases among healthy individuals are also reported. The virus induces cerebellar inflammation through its direct invasion into the brain, as many of them can pass BBB directly, while some of them disrupt the barrier. Activated microglia play a key role in mediating cerebellar inflammation responding to infectious agents, which causes the modulation of neuronal activity and would generate tremorogenic activity. We also briefly reviewed the mechanism of inflammation induced by CMV, DENV, INF, and SARS-CoV-2, and cerebellar inflammation associated with bacterial or fungal infection is very rare. Next, we introduced mtDNA and host autoimmunity as crucial factors for inducing cerebellar inflammation and related ataxia. Cell-mediated autoimmunity and the formation of autoantibodies are the distinct modes for immune-mediated ataxia. Furthermore, we discussed how ion channels modulate neurophysiological activity, including intrinsic excitability and excitatory and synaptic transmission. Potassium channels are the key regulator of the action potential of the Purkinje neurons in the cerebellum. SK channels are voltage-independent and modulate the compartment-specific dendritic plasticity. However, BK channels also contribute the spike repolarization and regulate the interspike interval. The dysfunction of these channels could be a reason for their motor dyscoordination and ataxia. Finally, we briefly reviewed the treatment strategy for acute cerebellar inflammation. Microglia play a crucial role in mediating inflammation, and the inhibition of its activation would be a promising treatment strategy. Blockade of potassium channels is very effective for limiting the activation of microglia, although it changes the activity of Purkinje neurons. Small compounds might develop to slow down the function of potassium channels rather than completely blocking, which may not interrupt the regularity of Purkinje cell activity.
